# Dabogratinib (TYRA-300), an FGFR3 Isoform-Selective Inhibitor: Preclinical and Initial Clinical Evidence of Antitumor Activity

**DOI:** 10.1158/1535-7163.MCT-25-0652

**Published:** 2025-10-13

**Authors:** Jacqueline H. Starrett, Eric L. Allen, Melissa Neal, Samhita Iyer, Qing Ye, Robert L. Hudkins, Viraj Degaonkar, Christine Lihou, Ronald V. Swanson, Valentina Boni, Jesús Fuentes-Antrás, Cedric Pobel, Yohann Loriot, Alena Zalutskaya, Erik Goluboff

**Affiliations:** 1 Tyra Biosciences, Carlsbad, California.; 2 NEXT Oncology, Hospital Universitario Quirónsalud Madrid, Madrid, Spain.; 3 Département d’Innovation Thérapeutique et des Essais Précoces (DITEP), Institut Gustave Roussy, Université Paris-Saclay, Villejuif, France.

## Abstract

Despite recent advances in the treatment of fibroblast growth factor receptor 3 (*FGFR3*)–altered metastatic urothelial carcinoma, there is no approved precision therapy that selectively targets FGFR3 while sparing other FGFR isoforms. Dabogratinib (TYRA-300)—a rationally designed selective FGFR3 inhibitor—was evaluated *in vitro* and *in vivo*. We also report three patient cases from the ongoing first-in-human, phase I/II SURF301 study (NCT05544552). Dabogratinib elicited a dose-dependent reduction in downstream signaling across three bladder cancer cell lines harboring an *FGFR3* fusion, mutation, or gatekeeper resistance mutation. In a xenograft model driven by an *FGFR3*^*S249C*^-activating mutation, dabogratinib treatment resulted in dose-dependent tumor growth inhibition with tumor regression observed at the highest doses. These preclinical findings are supported by the three case reports from the SURF301 study, which demonstrate early clinical activity in patients with advanced metastatic urothelial carcinoma with an *FGFR3* fusion or activating mutation.

## Introduction

Oncogenic alterations in fibroblast growth factor receptor 3 (*FGFR3*) have been found in many cancer types. They are seen most frequently in metastatic urothelial carcinoma (mUC; about 15%–20%) and are found in other solid tumor types, including breast, ovarian, endometrial, lung, pancreatic, gastric, renal, and colorectal cancers (1, 2). *FGFR3* mutations are also commonly found in non–muscle-invasive bladder cancer (NMIBC), including in ∼70% patients with intermediate-risk NMIBC (3, 4), and upper tract urothelial cancer, in up to 96% of patients with low-grade upper tract urothelial cancer (5, 6). The most common FGFR3 alteration is a point mutation in the ligand-binding domain, S249C, and the second most common is Y373C in the transmembrane domain (7). These two mutations account for approximately 80% of the FGFR3 mutations in bladder cancer, and like other cysteine substitutions in the extracellular and transmembrane domains (e.g., R248C and G370C), they act to constitutively activate the receptor via disulfide-mediated dimerization. Activating *FGFR3* gene fusions are also found in bladder cancer.

There are no approved FGFR3-selective targeted therapies in advanced/mUC and solid tumors harboring oncogenic *FGFR3* alterations. Several pan-FGFR inhibitors (e.g., erdafitinib, pemigatinib, and infigratinib) have demonstrated clinical benefit; however, response rates and duration of response are limited (8–10). Gatekeeper mutations are associated with resistance and were identified as having emerged while on treatment with infigratinib and other FGFR inhibitors (11, 12). The FGFR3 gatekeeper mutation occurs with a valine to methionine or, less commonly, leucine substitution at the V555 position of FGFR3, leading to a significant decrease in potency for all first-generation pan-FGFR inhibitors. We recently reported that erdafitinib was ineffective in a preclinical xenograft model harboring the V555M mutation (13). The emergence of acquired drug resistance may limit responses to treatment and presents a significant unmet need for patients.

The lack of specificity for FGFR3 may lead to FGFR1-, FGFR2-, and FGFR4-mediated side effects, such as hyperphosphatemia, diarrhea, and eye and nail toxicity. FGFR1 is expressed in kidney cells in which it regulates phosphate and calcium reabsorption, and its inhibition can cause hyperphosphatemia (14, 15). In a clinical trial of erdafitinib, hyperphosphatemia affected more than 70% of patients (8). A similarly high rate of FGFR-related toxicities has been reported in clinical studies of other nonselective FGFR inhibitors, including pemigatinib, infigratinib, and futibatinib (9, 10, 16).

Urothelial carcinoma is the ninth most common type of cancer diagnosed worldwide, with an estimated 613,791 people diagnosed in 2022 and 220,349 deaths (17). With the recent FDA approval of enfortumab vedotin in combination with pembrolizumab for the treatment of advanced mUC, this combination therapy has become the standard of care and is the preferred treatment choice in the first-line setting in the United States irrespective of mutation status (18). Despite recent advances in the treatment of *FGFR3*-altered mUC, the only approved pan-FGFR inhibitor after failure of at least one prior systemic therapy is erdafitinib, which is associated with significant toxicities driven by FGFR1/2/4 that limit its clinical utility. In mUC, erdafitinib showed an overall response rate of 35.3% with a median progression-free survival and overall survival of 5.6 and 12.1 months, respectively; however, the rate of serious adverse events (AE) was 41%, with toxicities that led to dose interruptions, reductions, and permanent drug discontinuation observed in 68%, 53%, and 21% of the patients, respectively (19). Some of these AEs are difficult to tolerate and manage (e.g., hyperphosphatemia, stomatitis, diarrhea, hand–foot syndrome, paronychia, blurred vision, and keratitis), which presents a significant challenge for patients. There is, therefore, a need for selective FGFR3 inhibitors to improve treatment outcomes and limit FGFR1/2/4-associated AEs.

Dabogratinib (TYRA-300) is a potential first-in-class, selective, oral, investigational FGFR3 inhibitor targeting oncogenic FGFR3 mutations, fusions, and gatekeeper resistance mutations (13). Here, we report dabogratinib preclinical data and initial first-in-human case reports of patients with *FGFR3*-altered mUC enrolled in the phase I portion of the ongoing SURF301 study (NCT05544552).

## Materials and Methods

### Cell lines and reagents

RT112/84 (RRID: CVCL_2714) and UM-UC-14 (RRID: CVCL_2747) cell lines were purchased from MilliporeSigma and authenticated by IDEXX BioAnalytics in 2020. Cell lines were routinely tested each month for *Mycoplasma* contamination using the MycoAlert Mycoplasma Detection Kit (Lonza). Cells were used within 20 passages after thawing. Synthego was contracted to genetically engineer CRISPR knock-in pools with the gatekeeper mutation V555M in the FGFR3 gene in RT112/84 cells. RT112/84 cells were clonally isolated from pooled edited cells and sequenced. A clone that contained the V555M knock-in mutation exclusively on the FGFR3::TACC3 fusion gene was selected. RT112/84 cells were cultured in RPMI-1640 (Gibco, #72400-047) media supplemented with 10% FBS. UM-UC-14 cells were cultured in minimum essential medium (Gibco, #41090-036) supplemented with 10% FBS, 1× GlutaMAX (Gibco, #35050-061), and 1× minimum essential medium nonessential amino acids (Gibco, #11140-050). All cells were cultured at 37°C and 5% CO_2_. The following primary antibodies were purchased from Cell Signaling Technology: total-Erk1/2 (tERK1/2; #4695, RRID: AB_390779), phospho-Erk1/2 T202/Y204 (pERK1/2; #4370, RRID: AB_2315112), and β-actin (#4970, RRID: AB_2223172). Erdafitinib was purchased from AstaTech (#40107). Dabogratinib (TYRA-300; 5-[(1R)-1-(3,5-dichloro-4-pyridyl)ethoxy]-3-[6-(2-methylsulfonyl-2,6-diazaspiro[3.3]heptan-6-yl)-3-pyridyl]-1H-indazole besylate salt) was synthesized as described by Hudkins and colleagues (13) and in the Supplementary Data file.

### Western blot analysis

The effects of dabogratinib on protein expression were examined by automated capillary electrophoresis for protein separation, followed by immunoassay-based detection using Bio-Techne’s Simple Western Jess machine according to their recommended protocol. RT112/84 and UM-UC-14 cells were seeded at 100,000 cells per well in a 96-well plate and allowed to adhere overnight. Prior to compound treatment, media were replaced with fresh media. Cells were treated with vehicle control (DMSO) or dabogratinib starting at 3 μmol/L and serially diluted fourfold for eight additional doses in duplicate. Cells were incubated with compound at 37°C and 5% CO_2_ for 2 hours. After treatment, cells were washed once with PBS (Gibco, #10010-023) at room temperature and then lysed in cold Bicine CHAPS lysis buffer (Protein Simple, #040-764) supplemented with 1× Halt Protease and Phosphatase Inhibitor (Thermo Fisher Scientific, #1861282) for 20 minutes. pErk1/2 was used at 1:50 dilution, and tERK1/2 and β-actin were used at 1:100 dilution. Data analysis was performed using Compass for Simple Western v6.1. AUC values for pERK and tERK are reported from the molecular weight chemiluminescence signal at 44 kDa spanning ± 10% either side using the standard dropped line settings.

### Mice

All animals were kept in pathogen-free microisolator housing under BSL2 guidelines approved by the CRADL Institutional Animal Care and Use Committee and in agreement with the NIH Guide for the Care and Use of Laboratory Animals. Mice were group housed on a 12-hour light/dark cycle with *ad libitum* access to water and standard mouse chow (Teklad 2920X). Athymic nude (Nu/Nu) mice from Charles River Laboratories (RRID: IMSR_CRL:088) were used for tumor growth inhibition (TGI) studies. Mice were 6 to 8 weeks old upon receipt.

### TGI xenograft studies

The human tumor cell line (UM-UC-14) was mixed 1:1 with Matrigel and injected subcutaneously into the flank of female athymic nude (nu/nu) mice (8 × 10^6^ cells per mouse). When tumors were established, mice were randomized to treatment groups according to tumor volume without blinding. Tumor sizes [tumor volume (mm^3^) = (a × b^2^/2), in which a represents the length and b represents the width of the tumor as determined by caliper measurements] and body weights were measured two or three times weekly. Time course of tumor growth is expressed as mean ± SEM. Percent TGI values were calculated based on the final tumor volume of the treatment group relative to the final tumor volume of the control group. Dabogratinib and erdafitinib were formulated in 30% hydroxypropyl-β-cyclodextrin.

### Clinical study

SURF301 (NCT05544552) is a phase I/II, open-label, international, multicenter dose-escalation and -expansion trial of dabogratinib, which was initiated to evaluate safety and preliminary antitumor activity of dabogratinib as a single agent in adults with *FGFR3*-altered advanced or mUC and other solid tumors. The primary objectives of the phase I portion of the study were to assess safety and tolerability and to identify the MTD and/or recommended phase II dose of dabogratinib as monotherapy. The key eligibility criteria included adults with Eastern Cooperative Oncology Group performance status of 0 to 1, adequate end-organ function, prior treatment with standard of care, baseline evaluable or measurable disease by investigator assessment per RECIST 1.1, and any histologically confirmed advanced solid tumors, including those harboring an oncogenic *FGFR3* mutation or fusion. Prior treatment with FGFR inhibitors was allowed but not required. Dabogratinib monotherapy was administered orally at 10 to 120 mg once daily and 40 and 50 mg twice daily in 28-day cycles. The protocol instructed patients to take study drug in a fasted state (1 hour before eating or 2 hours after eating). Intrapatient dose escalation was permitted. Disease response assessment was performed per RECIST v1.1 every 8 weeks. The AE assessment was done per The Common Terminology Criteria for Adverse Events v.5.0. The SURF301 trial is conducted in accordance with written standard operating procedures, in compliance with the Declaration of Helsinki and applicable global/local Good Clinical Practice regulations and International Council for Harmonization guidelines. At the time of this article, the study is ongoing.

### Preclinical statistical analysis

Data are presented as mean ± SEM. Differences between experimental groups were assessed using a Mann–Whitney U test. The significance threshold was set at *P* < 0.05. Statistical analyses were performed using GraphPad Prism (v10.3.1, RRID: SCR_002798).

## Results

### Pharmacodynamic profile of dabogratinib

**Figure 1.**
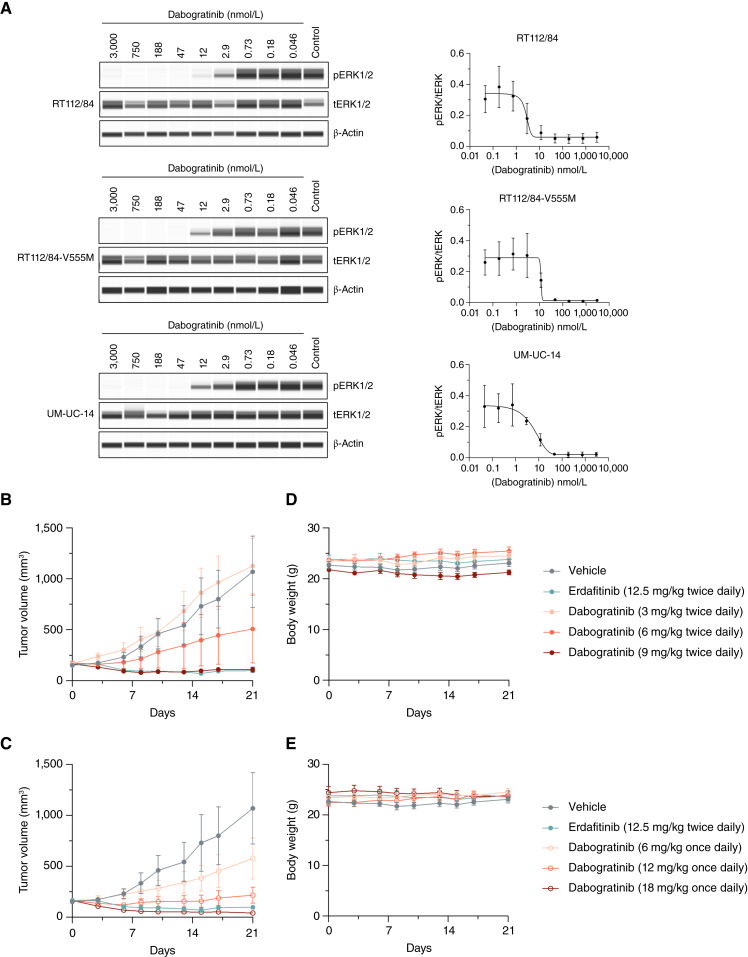
Dabogratinib demonstrates *in vitro* and *in vivo* efficacy in *FGFR3*-driven bladder cancer models. **A,** Assessment of downstream signaling after treatment for 2 hours with dabogratinib at the indicated dose or vehicle control in the indicated *FGFR3*-driven bladder cancer cell lines. One representative biological replicate is shown on the left and quantification of the ratio of pERK to tERK of *n* = 3 biological replicates is shown on the right. **B** and **C,** Antitumor activity in the *FGFR3*^S249C^-activating mutant UM-UC-14 bladder cancer xenograft model in nu/nu mice of various dosages of dabogratinib: (**B**) 3, 6, and 9 mg/kg twice daily and (**C**) 6, 12, and 18 mg/kg once daily and erdafitinib (12.5 mg/kg twice daily) shown in both **B** and **C**. **D** and **E,** Body weight averages for the dosage groups depicted in **B** and **C**, respectively. All dosages were by oral administration. Data in **B–E** were derived from the same study but split into separate graphs to enable visualization of each group. Data from vehicle- and erdafitinib-treated groups were repeated in **B** and **C**, as well as in **D** and **E**. Data points represent mean tumor volume (*n* = 6 per group, except 6 mg/kg twice daily dabogratinib dosing group in which one animal was found dead at day 7 of treatment, with *n* = 5) and error bars represent SEM.

The *in vitro* potency and selectivity of dabogratinib using kinase activity assays and cellular viability assays were previously reported by Hudkins and colleagues (13). Crystal structures with dabogratinib also demonstrated its ability to bind FGFR3 agnostic to the gatekeeper mutation residue (13). To explore the pharmacodynamic activity of dabogratinib, Western blot analysis of the downstream signaling pathway of FGFR3 was performed in three bladder carcinoma cell lines. RT112/84 is a bladder cancer cell line that contains an FGFR3::TACC3 fusion protein, and RT112/84-V555M contains the gatekeeper mutation V555M exclusively on the FGFR3::TACC3 fusion protein. UM-UC-14 is a bladder cancer cell line which harbors the *FGFR3*^S249C^ mutation. In the report by Hudkins and colleagues (13), cellular IC_50_ values for dabogratinib were 9 nmol/L in the RT112/84 cell line, 17 nmol/L in RT112/84-V555mol/L, and 16 nmol/L in UM-UC-14. Treatment with dabogratinib resulted in a dose-dependent decrease in the level of pERK1/2 in all three cell lines (Fig. 1A). Of note, a similar dose–response was observed for the gatekeeper-mutant cell line as for the two cell lines harboring only the activating alterations. These data confirm that treatment with dabogratinib elicits a dose-dependent reduction in downstream signaling of FGFR3 in bladder carcinoma cells.

### *In vivo* efficacy of dabogratinib

The *in vivo* activity of dabogratinib in the RT112/84 and RT112/84-V555M cell line–derived xenograft models, which contain the FGFR3::TACC3 fusion, was previously described (13). In this study, dabogratinib treatment resulted in tumor regression with a 12.5 mg/kg twice daily dosage across both models. To confirm the *in vivo* efficacy of dabogratinib in the context of a driver mutation (S249C) in *FGFR3* and explore the dose-dependent activity, the UM-UC-14 cell line–derived xenograft model was used. In this mouse model, dabogratinib was dosed orally once daily at 6, 12, and 18 mg/kg or twice daily at 3, 6, and 9 mg/kg for 21 days (Fig. 1B and C). No TGI was observed for dabogratinib at 3 mg/kg twice daily. Inhibition of tumor growth was observed for all other dabogratinib dosages tested: 6 mg/kg twice daily (53%), 9 mg/kg twice daily (90%), 6 mg/kg once daily (46%), 12 mg/kg once daily (80%), and 18 mg/kg once daily (96%). Although the 12 and 18 mg/kg once daily and 9 mg/kg twice daily dosages resulted in significant tumor volume reduction compared with the vehicle-treated group, the 3 and 6 mg/kg twice daily and 6 mg/kg once daily dosages were not statistically significant. Administration of dabogratinib once daily versus twice daily seemed to be more effective in the mouse model, with 18 mg/kg once daily resulting in the greatest reduction in tumor growth. We observed 91% TGI for 12.5 mg/kg twice daily erdafitinib; thus, dabogratinib (18 mg/kg once daily) resulted in more tumor regression than erdafitinib (12.5 mg/kg twice daily, *P* = 0.0244). There was no bodyweight loss observed in any of the treatment groups (Fig. 1D and E). These results demonstrate dose-dependent *in vivo* efficacy with tolerable once daily and twice daily dosages.

### First-in-human activity of dabogratinib monotherapy in patients with *FGFR3*-altered mUC

The safety and antitumor activity of dabogratinib as a monotherapy are being assessed in a first-in-human phase I/II clinical trial of dabogratinib (SURF301; NCT05544552) in patients with advanced or mUC and other solid tumors. Three single patient cases from the ongoing phase I portion of the study are described to illustrate early antitumor activity of dabogratinib monotherapy in patients with mUC harboring oncogenic *FGFR3* alterations. At the time of this publication, dosages through 100 mg once daily were cleared, and the optimal dabogratinib monotherapy dosage is yet to be established.

#### Case 1

**Figure 2.**
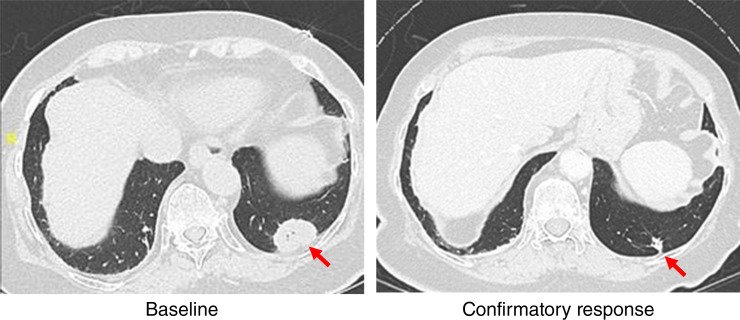
Dabogratinib demonstrates early clinical activity in a patient with mUC with an *FGFR3*^S249C^ mutation. CT images of a target lesion in the lung are shown at baseline and at 15.7 weeks as a confirmatory PR.

The patient was an 84-year-old female with stage IV urothelial carcinoma harboring an *FGFR3*^S249C^ mutation. Prior systemic therapies in the metastatic setting included gemcitabine with platinum chemotherapy (cisplatin and then carboplatin), followed by avelumab with unknown best overall response (BOR), docetaxel [BOR of partial response (PR)], and enfortumab vedotin (BOR of stable disease). Upon progression on enfortumab vedotin and passing required eligibility assessments per the study protocol, the patient was enrolled into the phase I portion of the SURF301 study. At baseline, the patient had one target lesion in the lung and nontarget lesions in the lung and bones. The patient has initiated dabogratinib monotherapy at the 90 mg dosage taken once daily continuously for 28-day cycles. The patient was later reduced to 60 mg once daily due to grade 2 peripheral sensory neuropathy that was deemed to be possibly related to the study drug in the opinion of the investigator. There were no grade ≥3 treatment-related AEs observed while on treatment. Radiologic disease assessment per RECIST v1.1 at 5.1 weeks showed an initial overall PR, with −55% change in the longest diameter of the target lesion compared with baseline. The response was confirmed on the subsequent imaging at 15.7 weeks with further tumor shrinkage of −64% versus baseline (Fig. 2). The patient has maintained response for 12.6 months and showed radiologic disease progression at 13.8 months of treatment. The patient remains on treatment after progression because of continuous clinical benefit from dabogratinib monotherapy.

#### Case 2

**Figure 3.**
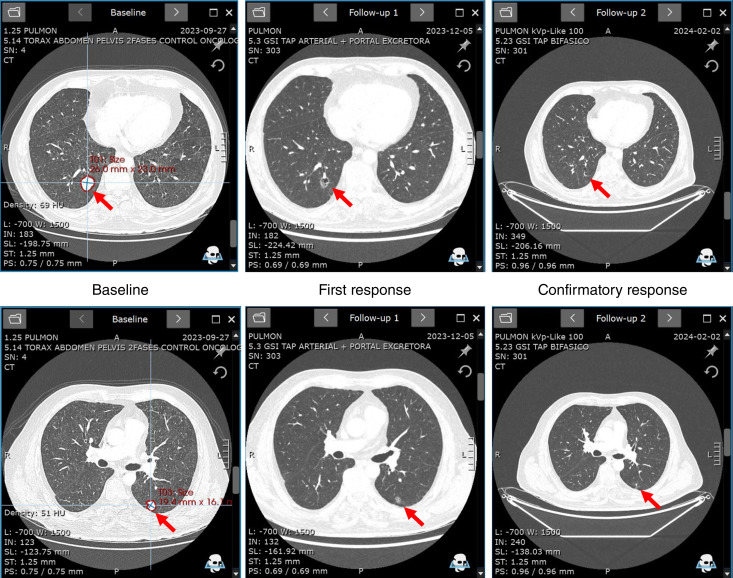
Dabogratinib demonstrates early clinical activity in a patient with mUC with an *FGFR3::JAKMIP1* fusion. CT images of target lesions in the lung are shown at baseline, first partial response at 8 weeks, and at 15.7 weeks as a confirmatory PR.

The patient was a 64-year-old male with stage IV urothelial carcinoma harboring *FGFR3::JAKMIP1* fusion (ch4: 1808661 and chr4: 6087356). Prior systemic therapies in the metastatic setting included gemcitabine, paclitaxel, carboplatin, and atezolizumab (BOR is unknown), followed by methotrexate, vincristine, and adriamycin (BOR is unknown). Upon progression on chemotherapy and passing required eligibility assessments per the study protocol, the patient was enrolled into the phase I portion of the SURF301 study. At baseline, the patient had four target lesions, including two in the lung and two in lymph nodes, and nontarget lesions in the lung and lymph nodes. The patient received dabogratinib monotherapy at 90 mg once daily continuously in 28-day cycles. The patient tolerated treatment well with no observed grade ≥3 AEs that were deemed to be related to the study drug in the opinion of the investigator. Radiologic disease assessment per RECIST v1.1 at 7.3 weeks showed an initial overall PR, with −33% change in the sum of the diameter of the target lesions compared with baseline. The response was confirmed on the subsequent imaging at 15.7 weeks with further decrease to −49% versus baseline (Fig. 3). The patient maintained a response for 7.4 months and showed radiologic disease progression at 9 months of treatment. The patient remains on treatment after progression because of continuous clinical benefit from dabogratinib monotherapy.

#### Case 3

**Figure 4.**
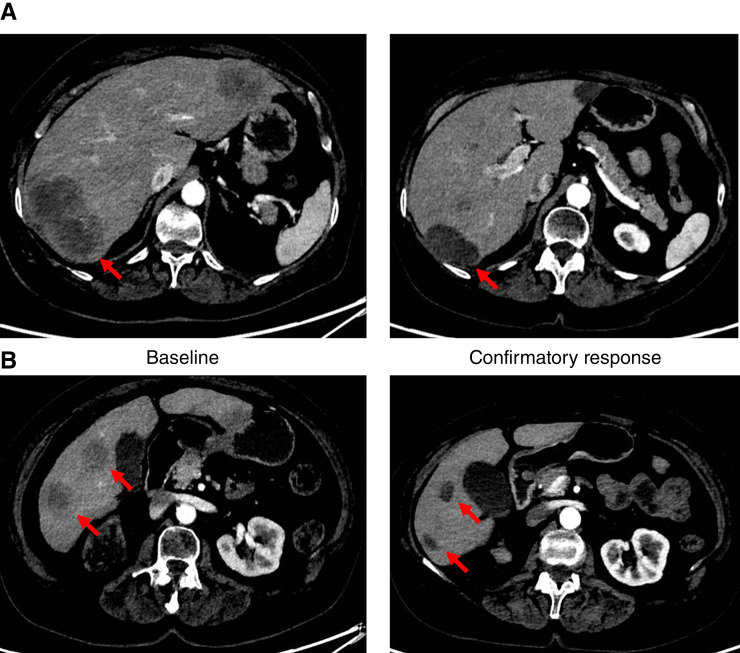
Dabogratinib demonstrates early clinical activity in a patient with mUC with an FGFR3::TACC3 fusion. CT images of a target lesion in the liver (**A**) and nontarget lesions in the liver (**B**) are shown at baseline and at 15.4 weeks as a confirmatory PR.

The patient was a 67-year-old female with stage IV urothelial carcinoma harboring an *FGFR3::TACC3* fusion. Prior systemic therapies in the metastatic setting included pembrolizumab with BOR of progressive disease, followed by enfortumab vedotin (BOR is unknown). Upon enfortumab vedotin treatment discontinuation due to disease progression and passing required eligibility assessments per the study protocol, the patient was enrolled into the phase I portion of the SURF301 study. At baseline, the patient had five target lesions, including two in the liver, two in the peritoneum, and one in the lymph node, and nontarget lesions in the liver. The patient has initiated dabogratinib monotherapy at 120 mg once daily continuously in 28-day cycles. After three cycles of treatment, the dose was reduced to 90 mg once daily because of grade 2 aspartate aminotransferase elevation that was deemed to be possibly related to the study drug in the opinion of the investigator. The patient tolerated treatment well, with no observed grade ≥3 AEs. Radiologic disease assessment per RECIST v1.1 at 7.4 weeks showed an initial overall PR, with a −46.2% change in the sum of the longest (or shortest for lymph nodes) diameter of the target lesions compared with baseline. The response was confirmed on the subsequent imaging at 15.4 weeks with a further decrease of −49.8% versus baseline (Fig. 4A and B). The patient maintained a response for 12 months and showed radiologic disease progression at 13.6 months of treatment. The patient remains on treatment after progression because of continuous clinical benefit from dabogratinib monotherapy.

## Discussion

In this report, we describe the preclinical activity and early clinical experience of dabogratinib, a novel, oral, selective inhibitor targeting oncogenic FGFR3 mutations, fusions, and gatekeeper resistance alterations. The goal of optimizing selectivity for FGFR3 is to enable effective inhibition of the target while avoiding the clinical symptoms that may emerge because of off-target inhibition of FGFR1, FGFR2, or FGFR4, which were observed in patients treated with pan-FGFR inhibitors in the clinic (8–10, 16). In preclinical studies, dabogratinib has demonstrated similar *in vitro* potency in *FGFR3*-driven cell lines as pan-FGFR inhibitors (13). However, although IC_50_ values remain low for erdafitinib, futibatinib, pemigatinib, and infigratinib in FGFR1-, FGFR2-, and FGFR4-driven Ba/F3 cell lines, dabogratinib was less potent for these isoforms, translating to 19-fold selectivity for FGFR3 over FGFR2, 63-fold selectivity over FGFR1, and 55-fold selectivity over FGFR4. In the current study, with a direct comparison of dabogratinib and erdafitinib *in vivo* in the UM-UC-14 xenograft model, dabogratinib (18 mg/kg once daily) resulted in slightly more tumor regression than erdafitinib (12.5 mg/kg twice daily).

In addition to improving upon selectivity for FGFR3, dabogratinib also addresses pan-FGFR inhibitors’ lack of efficacy against gatekeeper resistance mutations, which have been detected in tumors while on treatment with pan-FGFR inhibitors (11, 12). FGFR3 signals through the MAPK pathway to promote tumor proliferation and survival in the context of cancer (14). The pharmacodynamic effects of dabogratinib on downstream signaling were demonstrated by Western blot analysis of pERK1/2, indicating that dabogratinib reduced FGFR3 signaling dose-dependently in the context of an FGFR3-activating mutation, fusion, or gatekeeper resistance mutation. Although pan-FGFR inhibitors have similarly reported reduction in pERK1/2 in FGFR3 fusion or FGFR3-amplified cancer cell lines (15, 20, 21), this is the first report of similar pERK1/2 reduction in a bladder cancer cell line harboring the gatekeeper resistance mutation, which has important implications for the emergence of on-target resistance. Importantly, in a previous study, dabogratinib maintained efficacy in the RT112/84-V555M xenograft model *in vitro* and *in vivo*, whereas erdafitinib lost activity in the RT112/84-V555M cell line *in vitro* and was unable to cause tumor regression *in vivo* (13). Dabogratinib achieved similar levels of tumor regression in both the RT112/84 xenograft in the previous study and in the UM-UC-14 xenograft in the current study, displaying *in vivo* efficacy across FGFR3 fusions, point mutations, and in the context of an FGFR3 gatekeeper mutation. Therefore, dabogratinib has the potential to improve upon existing pan-FGFR inhibitors by increasing selectivity for FGFR3, which reduces the potential for off-target toxicities, as well as maintaining efficacy in the presence of common resistance mechanisms.

Early clinical cases support our preclinical findings. Preliminary safety and efficacy data reported with dabogratinib demonstrated six confirmed PRs in 11 (54.5%) *FGFR3*-altered mUC efficacy-evaluable patients at dosages ≥ 90 mg once daily, with a very low frequency of grade 3 treatment-related AEs (22). The patient cases described here provide encouraging early antitumor activity as evident by confirmed and durable responses observed in heavily pretreated patients with mUC harboring an activating *FGFR3* mutation in one case and an *FGFR3* fusion in the two others. The median progression-free survival for erdafitinib is 5.6 months (19). The patient reported here with an *FGFR3*^S249C^ mutation had a response for 12.6 months. Erdafitinib has shown better efficacy in patients with *FGFR3* mutations versus fusions with overall response rates of 49% and 16%, respectively, with the response to the most common *FGFR3::TACC3v1* fusion being 36% (8)**.** The patient reported here with the *FGFR3::TACC3* fusion has maintained a response for 12 months, and the patient with the *FGFR3::JAKMIP1* fusion responded to dabogratinib monotherapy and maintained a response for 9 months, suggesting that dabogratinib may address unmet medical need in patients with *FGFR3* fusions in addition to those harboring *FGFR3* mutations. Erdafitinib is also associated with a high frequency of grade 3 to 4 treatment-related AEs (8). No treatment-related grade ≥ 3 toxicities were observed in these three patients.

Taken together, reported preclinical and early clinical case data support further dabogratinib development in the clinic given its potential to address two key limitations of existing therapies: (1) selectivity for FGFR3 and (2) activity against the gatekeeper resistance mutation. The SURF301 trial in mUC continues to advance, and dabogratinib will also be studied in the phase II SURF302 trial in *FGFR3*-altered patients with intermediate-risk NMIBC.

## Supplementary Material

Supplementary Data Supplementary Data file describes the structure and synthesis method for dabogratinib.

## Data Availability

The data generated in this study are available upon request from the corresponding author.
