# Supplementary material for: Dabogratinib (TYRA-300), an FGFR3 Isoform-Selective Inhibitor: Preclinical and Initial Clinical Evidence of Antitumor Activity
Source: Mol Cancer Ther. 2025 Oct 13;25(3):408–15. doi: 10.1158/1535-7163.MCT-25-0652 (PMC13060625; doi:10.1158/1535-7163.MCT-25-0652)
Supplement: Supplementary Data — file describes the structure and synthesis method for dabogratinib. [file mct-25-0652_supplementary_data_suppsd.docx]

Supplementary Data

Dabogratinib (5-[(1R)-1-(3,5-dichloro-4-pyridyl)ethoxy]-3-[6-(2-methylsulfonyl-2,6-diazaspiro[3.3]heptan-6-yl)-3-pyridyl]-1H-indazole) and its preparation, are described in International Patent Application No. PCT/US2021/065679 (WO2022/147246), US 12,264,149 and *J. Med. Chem*. **2024**, *67*, 16737−16756 (13).

5-[(1R)-1-(3,5-Dichloro-4-pyridyl)ethoxy]-3-[6-(2-methylsulfonyl-2,6-diazaspiro[3.3]heptan-6-yl)-3-pyridyl]-1H-indazole besylate.

Step 1. 5-[(1R)-1-(3,5-dichloro-4-pyridyl)ethoxy]-3-(6-fluoro-3-pyridyl)-1-tetrahydropyran-2-yl-indazole. A mixture of 5-[(1R)-1-(3,5-dichloro-4-pyridyl)ethoxy]-3-iodo-1-tetrahydropyran-2-yl-indazole (25.0 g, 48.35 mmol, 1.0 equiv), 2-fluoro-5-(4,4,5,5-tetramethyl-1,3,2-dioxaborolan-2-yl)pyridine (12.94 g, 58.02 mmol, 1.2 equiv), potassium carbonate (13.34 g, 96.70 mmol, 2.0 equiv) and [1,1’-bis(diphenylphosphino)ferrocene]-dichloropalladium(II) (3.56 g, 4.84 mmol, 0.1 equiv) in 1,4-dioxane (250 mL) and water (25 mL) was sparged with nitrogen for 15 minutes and then heated at 100 °C for 4 hours. After cooling to room temperature, the reaction mixture was filtered over a Celite bed (250 g). The Celite bed was washed with ethyl acetate (100 mL) and the combined filtrate was concentrated under reduced pressure. The residue was dissolved in dichloromethane (50 mL) and purified over a silica plug (500 g), eluting with 20% ethyl acetate in heptanes. Product containing fractions were pooled and concentrated to dryness under reduced pressure. The resulting light brown solid was triturated in acetonitrile (50 mL) at room temperature for 1 hour. The solids were vacuum filtered to give a white solid (16.77 g, 71% yield). LCMS m/z = 487 (M+H).

Step 2. 5-((R)-1-(3,5-Dichloropyridin-4-yl)ethoxy)-3-(6-(6-(methylsulfonyl)-2,6-diazaspiro[3.3]heptan-2-yl)pyridin-3-yl)-1-(tetrahydro-2H-pyran-2-yl)-1H-indazole. A mixture of 5-[(1R)-1-(3,5-dichloro-4-pyridyl)ethoxy]-3-(6-fluoro-3-pyridyl)-1-tetrahydropyran-2-yl-indazole (16.77 g, 34.5 mmol, 1.0 equiv), 2-(methylsulfonyl)-2,6-diazaspiro[3.3]heptane dimesylate (15.23 g, 41.40 mmol, 1.2 equiv) and potassium carbonate (14.28 g, 103.50 mmol, 3.0 equiv) in 1-methyl-2-pyrrolidone (160 mL) was heated at 120 °C for 16 hours. The reaction mixture was cooled to room temperature, followed by the addition of water (200 mL). The resulting suspension was stirred at room temperature for 1 hour then vacuum filtered. After drying the solids under vacuum at room temperature for 1 hour, the solids were suspended in water (250 mL). The suspension was stirred at room temperature for 1 hour. The solids were vacuum filtered to give an off white solid (20.7 g, 93% yield) after drying under vacuum at 50 °C for 16 hours. LCMS m/z = 643.1 (M+H).

Step 3. (R)-5-(1-(3,5-Dichloropyridin-4-yl)ethoxy)-3-(6-(6-(methylsulfonyl)-2,6-diazaspiro[3.3]heptan-2-yl)pyridin-3-yl)-1H-indazole. Trifluoroacetic acid (210 mL, 2745 mmol, 85.0 equiv) was added over 20 minutes, while keeping the internal temperature below 15 °C to a solution of 5-((R)-1-(3,5-dichloropyridin-4-yl)ethoxy)-3-(6-(6-(methylsulfonyl)-2,6-diazaspiro[3.3]heptan-2-yl)pyridin-3-yl)-1-(tetrahydro-2H-pyran-2-yl)-1H-indazole (20.61 g, 32.1 mmol, 1.0 equiv) in dichloromethane (210 mL) at 5 °C. The resulting solution was warmed to room temperature for 30 minutes and then stir at room temperature for 5 hours. The volatiles were removed under reduced pressure to give a brown oil. The residue was diluted with dichloromethane (200 mL) and poured over a slurry of crushed ice (200 g) and water (100 mL). The mixture was treated with sodium bicarbonate (200 g) in several portions to adjust the pH to 8-9. The resulting suspension was vacuum filtered, and the filtrate was transferred to a separatory funnel. The mixture was diluted with saturated sodium bicarbonate (100 mL). The organic layer was separated, and the aqueous layer was extracted with dichloromethane (2 x 100 mL). The combined organic layers were concentrated to dryness under reduced pressure to give a light brown solid (23.0 g). This reaction was repeated, and the combined crude material (46.1 g) was suspended in acetonitrile (460 mL) and heated at 50 °C for 3 hours. The suspension was cooled to room temperature and stirred for 12 hours. The solids were collected and dried then further purified by trituration in methanol (35 mL) for 1 hour to give an off white solid (25.3 g, 70%, 99.3% HPLC purity). LCMS m/z = 559.2 (M+H); 1H NMR (400 MHz, DMSO-d6) δ 13.02 (br s, 1H), 8.59 (s, 2H), 8.52 (dd, J = 0.6, 2.2 Hz, 1H), 7.87 (dd, J = 2.4, 8.6 Hz, 1H), 7.46 (d, J = 8.9 Hz, 1H), 7.16 (d, J = 2.1 Hz, 1H), 7.09 (dd, J = 2.3, 9.0 Hz, 1H), 6.54 (dd, J = 0.4, 8.6 Hz, 1H), 6.10 (q, J = 6.6 Hz, 1H), 4.17 (s, 4H), 4.12 (s, 4H), 3.03 (s, 3H), 1.76 (d, J = 6.6 Hz, 3H).

A suspension of 22 (5.0 g, 8.94 mmol, 1 equiv) in acetone (180 mL) was treated with a solution of benzenesulfonic acid (1.63 g, 10.32 mmol, 1.15 equiv) in acetone (17 mL) at room temperature. After 16 hours, the precipitate was filtered, washed with a 1 to 1 mixture of acetone and ethyl ether (3 x 20 mL) and dried at 50 °C for 5 hours and at room temperature for 2.5 days under vacuum to give an off-white solid (6.28 g, 98% yield). The suspension of TYRA-300 besylate (10.03 g, 13.98 mmol) in ethyl alcohol (1 L) was heated with stirring at 67 °C under a nitrogen atmosphere. After 16 hours, the suspension was cooled to room temperature without stirring for 30 minutes, filtered, washed with cold ethyl alcohol (3 x 120 mL) and dried at 50 °C for 5 hours and at room temperature for 16 hours under vacuum to give a white solid (8.35 g, 83% yield, 99.5% HPLC purity). LCMS m/z = 559.1 (free base, M+H); 1H NMR (400 MHz, DMSO-d6) δ = 13.27 (br s, 1H), 8.58 (s, 2H), 8.36 (d, J = 1.7 Hz, 1H), 8.24 (br d, J = 9.2 Hz, 1H), 7.65 - 7.56 (m, 2H), 7.51 (d, J = 9.2 Hz, 1H), 7.36 - 7.24 (m, 3H), 7.21 (d, J = 2.2 Hz, 1H), 7.13 (dd, J = 2.3, 9.0 Hz, 1H), 6.94 (br d, J = 8.8 Hz, 1H), 6.14 (q, J = 6.7 Hz, 1H), 4.42 (s, 4H), 4.15 (s, 4H), 3.04 (s, 3H), 1.77 (d, J = 6.7 Hz, 3H).
